# Microbial synthesis of core/shell gold/palladium nanoparticles for applications in green chemistry

**DOI:** 10.1098/rsif.2012.0003

**Published:** 2012-03-07

**Authors:** Kevin Deplanche, Mohamed L. Merroun, Merixtell Casadesus, Dung T. Tran, Iryna P. Mikheenko, James A. Bennett, Ju Zhu, Ian P. Jones, Gary A. Attard, J. Wood, Sonja Selenska-Pobell, Lynne E. Macaskie

**Affiliations:** 1 Unit of Functional Bionanomaterials, School of Biosciences, University of Birmingham, Edgbaston, Birmingham B15 2TT, UK; 2 School of Metallurgy and Materials, University of Birmingham, Edgbaston, Birmingham B15 2TT, UK; 3 School of Chemical Engineering, University of Birmingham, Edgbaston, Birmingham B15 2TT, UK; 4 Institut für Radiochemie, Forschungszentrum Dresden Rossendorf, PO Box 510119, 01314 Dresden, Germany; 5 School of Chemistry, Cardiff University, Park Place, Cardiff CF10 3AT, UK

**Keywords:** bioreduction, bimetallic catalysts, core/shell, *Escherichia coli*, gold, palladium

## Abstract

We report a novel biochemical method based on the sacrificial hydrogen strategy to synthesize bimetallic gold (Au)–palladium (Pd) nanoparticles (NPs) with a core/shell configuration. The ability of *Escherichia coli* cells supplied with H_2_ as electron donor to rapidly precipitate Pd(II) ions from solution is used to promote the reduction of soluble Au(III). Pre-coating cells with Pd(0) (bioPd) dramatically accelerated Au(III) reduction, with the Au(III) reduction rate being dependent upon the initial Pd loading by mass on the cells. Following Au(III) addition, the bioPd–Au(III) mixture rapidly turned purple, indicating the formation of colloidal gold. Mapping of bio-NPs by energy dispersive X-ray microanalysis suggested Au-dense core regions and peripheral Pd but only Au was detected by X-ray diffraction (XRD) analysis. However, surface analysis of cleaned NPs by cyclic voltammetry revealed large Pd surface sites, suggesting, since XRD shows no crystalline Pd component, that layers of Pd atoms surround Au NPs. Characterization of the bimetallic particles using X-ray absorption spectroscopy confirmed the existence of Au-rich core and Pd-rich shell type bimetallic biogenic NPs. These showed comparable catalytic activity to chemical counterparts with respect to the oxidation of benzyl alcohol, in air, and at a low temperature (90°C).

## 1. Introduction

Until recently, only chemical and physical synthesis methods were available to produce metallic nanoparticles (NPs) but increasing pressure to develop ‘clean’ nanomaterial synthesis methods has led to a growing interest in biotransformations as a route to controlled growth of nanoscale structures. As a result, the design, synthesis and characterization of biologically synthesized and stabilized NPs have recently become areas of significant interest. Advantages of using micro-organisms as nanofactories are multiple, being environmentally benign and often cheaper than chemical methods. Biological synthesis of NPs is scalable, offers particle size and shape control, and can even be coupled to the remediation of precious metal-containing wastes [1]. Various biological alternative approaches to the manufacture of supported metal clusters have been developed. Biotemplating, a ‘bottom-up’ approach, uses highly ordered biomolecules such as DNA and proteins (e.g. microtubules, bacterial S-layer proteins, flagellin) to grow metal clusters [2–4]. Bioreductive routes use the ability of some bacterial cells to reduce metal precursors enzymatically to the zero-valent state via an electron donor [5,6], usually leading to the formation of metallic NPs at the cell surface that exhibit a catalytic behaviour similar or superior to metallic NPs prepared using chemical methods in a wide range of reactions [7–11].

Bimetallic NPs that exhibit a core/shell structure [12] were recently shown to possess increased catalytic activity in several reactions [13–15]. For example, the tuned coverage of gold (Au) NPs by small platinum entities was found to maximize Pt efficacy in a fuel cell electrocatalyst [13], while the enhanced utility of palladium (Pd)–Au bimetallic was shown for selective oxidation reactions [14,15]. Although many physico-chemical techniques have been devised to prepare Pd–Au nanomaterials, the synthesis of non-random Pd–Au alloys with highly ordered controlled atomic distribution remains elusive. The structure of bimetallic combinations, which is dictated by the preparation conditions, is crucial in order to obtain the necessary synergistic interactions that lead to increases in catalytic activity. This work presents a simple, facile, two-step biochemical and chemical hybrid route to produce ordered Au–Pd core/shell nanostructures with strong catalytic activity in the oxidation of benzyl alcohol.

## 2. Material and methods

### 2.1. Organisms and culture conditions

*Escherichia coli* MC4100 (provided by Prof. J. A. Cole, University of Birmingham, UK) was maintained aerobically at 30°C on nutrient agar plates (Oxoid Ltd, Basingstoke, UK). Precultures (10% inoculum (v/v)) from a mid-exponential phase culture grown anaerobically in nutrient broth no. 2 (NB no. 2 (Oxoid, UK) containing 50 mM sodium formate) were grown overnight at 37°C in NB no. 2 under anaerobic respiratory conditions (NB no. 2 supplemented with 0.4% sodium fumarate (w/v) and 0.5% glycerol (v/v), final concentrations) to maximize hydrogenase expression [11].

For Pd(II) and Au(III) bioreduction experiments, cultures of *E. coli* MC4100 were grown as above in 2 l Durham bottles almost filled to the brim with medium (NB no. 2) and sealed with rubber stoppers. Mid-logarithmic phase cultures (OD_600_ = 0.5–0.7) were harvested by centrifugation (12 000*g*, 15 min), washed three times in 100 ml of degassed 3-(N-morpholino)propanesulphonic acid–NaOH buffer (20 mM, pH 7.2), resuspended in 50 ml of the same buffer and stored at 4°C as concentrated cell suspensions until use, usually the next day. Cell concentration (mg ml^−1^) was determined by correlation to a pre-determined OD_600_ to dry weight conversion.

### 2.2. Pd(II) and Au(III) solutions

For the preparation of Pd(0)-coated cells (bioPd), an aqueous Pd(II) solution (2 mM, to pH 2.3 with 0.01 M HNO_3_) was made by dissolving an appropriate amount of sodium tetrachloropalladate (Na_2_PdCl_4_, Sigma-Aldrich, Poole, UK). Similarly, aqueous Au(III) solutions (1 mM, to pH 2.3 with 0.01 M HNO_3_) were made by dissolving hydrogen tetrachloroaurate (HAuCl_4_·*n*H_2_O, Sigma-Aldrich) in pre-acidified distilled water.

### 2.3. Manufacture of biomass-supported palladium/gold nanoparticles

First, *E. coli* cells were palladized for examination as follows. A known volume of concentrated resting cell suspension (see §2.1) was transferred anaerobically into 200 ml serum bottles and an appropriate volume of degassed 2 mM Pd(II) solution was added so that the final ratio (weight of Pd:dry weight of cells) was 1:19, giving the loading of 5% (w/w) Pd on biomass. Cells/Pd mixtures were left to stand (30 min, 30°C) before H_2_ was sparged through the suspension (200 ml min^−1^; 20 min). During H_2_ sparging, the colour of the cell/Pd mixtures went from yellow to grey, indicating the reduction of cell surface-bound Pd(II) into Pd(0). Complete removal of Pd(II) from supernatants was confirmed by the SnCl_2_ assay (see §2.4). Next, bioPd was recovered by centrifugation (12 000*g*, 15 min), washed three times in distilled water and resuspended in distilled water so that the final ratio of Au(III) solution to bioPd suspension was 4:3 (v/v). The bioPd suspension was degassed (20 min) and transferred anaerobically into an appropriate volume of H_2_-saturated Au(III) solution (by sparging H_2_ in the solution for 30 min at 200 ml min^−1^) so that the final ratio of Pd:Au was 1:1 by mass. The bioPd–Au(III) mixture was allowed to react overnight in a rotary shaker (150 r.p.m., 30°C) and supernatants were assayed for residual Pd(II) and Au(III) (see §2.4) to ensure complete removal of both metal species. The final material was recovered as above, washed three times in distilled water, once in acetone and left to dry in air.

### 2.4. Assay of soluble Au(III) and Pd(II)

During bioPd manufacture, complete removal of Pd(II) from solution was confirmed by assaying cell/Pd mixture supernatants for residual Pd(II) spectrophotometrically (SnCl_2_ method [16]). Removal of Au(III) from test solutions was monitored by the thiamine–phloxine assay [17].

### 2.5. Electron microscopy and energy dispersive X-ray analysis of Pd–Au-loaded biomass

Following metal deposition, pellets of metal-loaded bacteria were prepared for transmission electron microscopy (TEM). Preparations were rinsed twice with distilled water, fixed in 2.5% (w/v) glutaraldehyde, centrifuged, resuspended in 1.5 ml of 0.1 M cacodylate buffer (pH 7) and stained in 1% osmium tetroxide in 0.1 M phosphate buffer, pH 7 (60 min). Cells were dehydrated using an ethanol series (70%, 90%, 100%, 100%, 100% dried ethanol, 15 min each) and washed twice in propylene oxide (15 min, 9500*g*). Cells were embedded in epoxy resin and the mixture was left to polymerize (24 h; 60°C). Sections (100–150 nm thick) were cut from the resin block, placed onto a copper grid and viewed with a JEOL 1200CX2 TEM; accelerating voltage 80 keV.

For energy dispersive X-ray (EDX) microanalysis, metallized cells were dispersed in water and then deposited on a carbon thin film coating copper TEM grids (Agar Scientific, grid thickness: 20–30 nm). A field emission gun Tecnai F20 microscope operating at 200 kV was used for high-resolution (HR) STEM imaging. In the STEM mode, the smallest condenser aperture C2 = 3 μm (which lowers the electron dose as well as reducing the beam damage) was used; the C1 lens was set to spot size 8; the electron probe size was less than 1 nm; and the camera length was 150 mm, chosen to minimize noise and artefacts caused by diffraction contrast and bright-field electrons. An X-Max Silicon Drift Detector (SDD) was attached to the microscope to perform EDX element mapping in STEM mode**.** This SDD has an active area of 80 mm^2^, which is advantageously large to produce high X-ray count-rates, reduce the acquisition time needed and thus ease the drift problems of nanoscale objects. The specimen was tilted to 18° to maximize the X-ray collection of the detector. Detector controlling, analysing and processing were performed using the INCA software, which also provides the SiteLock function to automatically correct the drift of the particles.

### 2.6. Surface characterization of biomass-supported palladium/gold nanoparticles by cyclic voltammetry

The NP surface initially presented an organic layer that was removed by suspending the powder samples in 6 M NaOH (p.a. grade) solution for six weeks, changing the solution every 2 days and rinsing with ultrapure water. Subsequently, the cleaned NPs were kept in a suspension with ultrapure water. All electrochemical experiments were performed in an electrochemical cell as described previously [18]. All electrolytes were prepared using 18.2 M*Ω* cm Milli-Q water and Suprapur (Aristar) grade H_2_SO_4_. Electrolyte solutions were degassed (30 min) before each experiment using oxygen-free nitrogen. A Pd–hydrogen reference electrode was used in all experiments; all data were given in reference to this electrode unless otherwise stated. Data acquisition was carried out as described previously [18]. For the electrochemical measurements, 10 µl of a cleaned NP suspension was deposited on a glassy carbon support electrode. The NPs were allowed to deposit under gravity on top of the electrode and allowed to dry in air to form a homogeneous layer. After the NPs were dried, the electrode was gently rinsed with ultrapure water. The electrolyte solution for electrochemical characterization consisted of 0.1 M H_2_SO_4_. All surface characterization measurements were performed at 100 mV s^−1^ and the potential range was from 0.1 to 1.5 V (versus Pd–hydrogen) unless otherwise stated.

### 2.7. X-ray absorption spectroscopic characterization of palladium/gold nanoparticles

Palladium K-edge and gold L_III_-edge X-ray absorption spectra were collected at the Rossendorf Beamline (ROBL) located at the European Synchrotron Radiation Facility (ESRF), Grenoble, France, using a Si(111) double-crystal monochromator and Si-coated mirrors for focusing and rejection of higher harmonics. Data were collected at room temperature in transmission or in fluorescence mode using an argon (Ar)-flushed ionization chamber or a 13-element Ge detector, respectively. The energies were calibrated by measuring the Pd K-edge and Au L_III_-edge transmission spectra of Pd and Au foil and defining the first inflection point as 24 350 and 11 919 eV, respectively. The Pd–Au-loaded sample was measured as dry sample (powder). The extended X-ray absorption fine structure (EXAFS) oscillations were isolated from the raw, averaged data by removal of the pre-edge background, approximated by a first-order polynomial, followed by µ_0_-removal via spline-fitting techniques and normalization using a Victoreen function. Dead-time correction was applied to fluorescence data. The amplitude reduction factors were obtained to be 0.88 for Pd and 0.92 for Au by fits of the referenced foils, and fixed in the analysis of the EXAFS spectra. The shift in threshold energy, *ΔE*_0_, was varied as a global parameter in the fits. The theoretical scattering phase and amplitude functions used in data analysis were calculated using the FEFF8 [19]. For the Pd edge EXAFS spectra, data for phase-shifts and backscattering amplitudes were obtained from the PdO (Pd–O scattering) and Pd foil (Pd–Pd scatterings) reference compounds. The Au–Pd reference file used for fitting the Au edge EXAFS spectrum of the experimental sample was determined by a theoretical calculation.

During the fits of EXAFS spectra and in order to reduce the number of fitted variables, the following constraints were applied:

— The bond distance and Debye–Waller factors for Pd–Au bonds were constrained to be equivalent for both Pd and Au edges.
— The ratio of coordination numbers of Pd–Au and Au–Pd pairs must be related to the overall composition of Au and Pd in the sample: *N*_AB_ = *x*_B_/*x*_A_ × *N*_BA_, where A and B are the absorbing atoms and *x*_A_ and *x*_B_ are the molar concentrations of Pd and Au in the sample [20].

### 2.8. Testing of catalytic activity of the palladium/gold nanoparticles on dried bacteria

Samples prepared as above (here to 2.5% Pd/2.5% Au (w/w) on the bacteria) were tested for catalytic activity in a 50 ml Parr 4592 batch reactor loaded with 50 ml benzyl alcohol and 180 mg catalyst. The reactor was sealed and allowed to reach 90°C before pressurizing with air (6 bar), maintained at a constant value by continually feeding air. Samples were periodically removed using a sample valve, filtered (0.2 μm) and then analysed using a Fisons GC8000/MD800 GC/MS versus commercial standards.

## 3. Results and discussion

**Figure 1.**
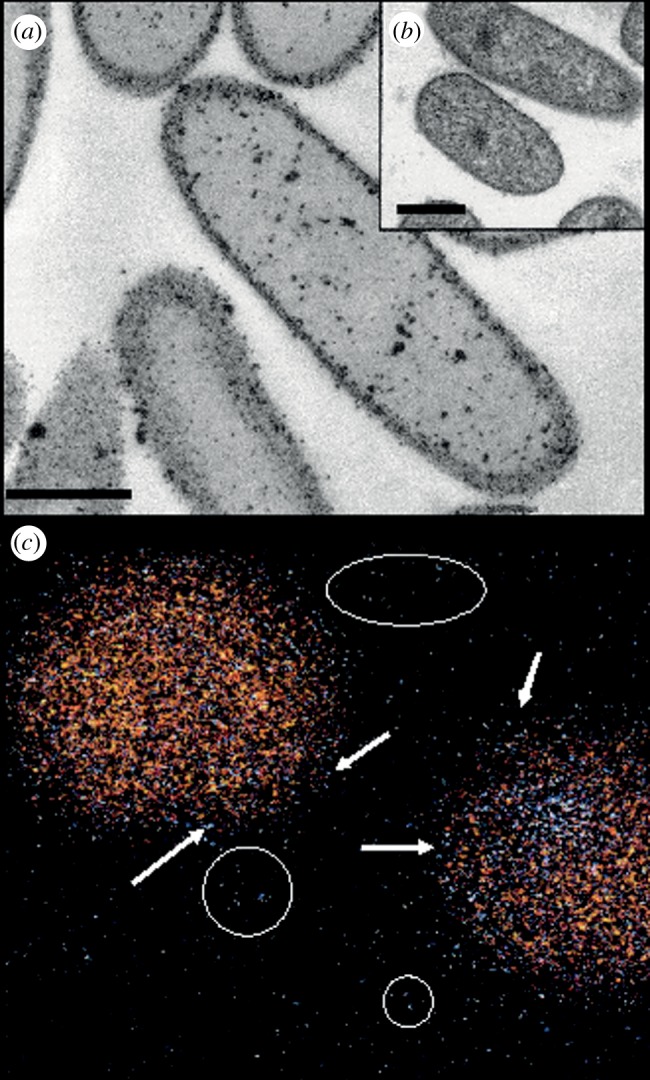
Electron microscopy of metallized cells of *E. coli* MC4100. (*a*) TEM of cells of *E. coli* MC4100 following the sequential reduction of Pd(II) and Au(III) (5%/5% Pd–Au on biomass w/w); untreated cells are shown in inset (*b*). Scale bars are 500 nm. (*c*) EDX mapping of two Pd–Au particles showing superimposed Au and Pd distributions: yellow, X-ray signal intensity from the characteristic *L*_α_ transitions of Au; blue, the characteristic *L*_α_ transitions of Pd. The particle on the right-hand side has segregation between Pd and Au with a clearly observed Pd-rich region. The particle on the left-hand side shows homogeneous mixing between Pd and Au. Regions of Pd are apparent at the surface of the nanoparticles (arrowed) and some areas between the NPs also indicated the presence of Pd (circled). Individual distributions of Au and Pd, together with complementary high-angle annular dark field microscopy, which provides atomic number contrast, were described previously [21].

We present a facile, size-controlled and cost-efficient method to synthesize Pd–Au core/shell nanostructures using *E. coli* which, in contrast to *Desulfovibrio desulfuricans* [21], can grow to high density at scale, and does not produce H_2_S, a catalyst poison. NP synthesis relies on the ability of *E. coli* cells to reduce Pd(II) ions enzymatically from a precursor (PdCl_4_^2−^ salt) using H_2_ as an electron donor [11]. We postulated that pre-palladizing cells with a fine layer of Pd(0) would lead to an increase in the rate of Au(III) reduction under H_2_ (see electronic supplementary material, figure S4) and result in the incorporation of Au atoms into the Pd seeds. The combined use of imaging and bulk/surface probing techniques permits detailed molecular- and atomic-scale structural analysis of the biomass-supported Pd–Au nanostructures. Following the sequential reduction of Pd(II) and Au(III), *E. coli* cells exhibited complete coverage of both the cell surface and the periplasmic space, with some cells showing a small number of intracellular NPs (figure 1*a*). Some large clusters were observed (figure 1*a*); a bimodal size distribution was reported previously for chemically synthesized Pd–Au NPs [22] and for bioNPs on *D. desulfuricans* [21]. Bimetallic Pd–Au particles of approximately 16 nm were examined with respect to their Au and Pd distributions as shown by the characteristic X-ray signals from *L*_α_ transitions for Au and Pd atoms. In accordance with other work using high-angle annular dark field analysis [21], which produces image contrast dependent on atomic number, figure 1*c* shows that Pd agglomerates (arrowed) decorate an Au-rich NP core region, with some Pd detected also between the NPs (encircled). Analysis by X-ray powder diffraction (electronic supplementary material, figure S5) [23] showed clearly crystalline Au(0), but not Pd(0), components.

**Figure 2.**
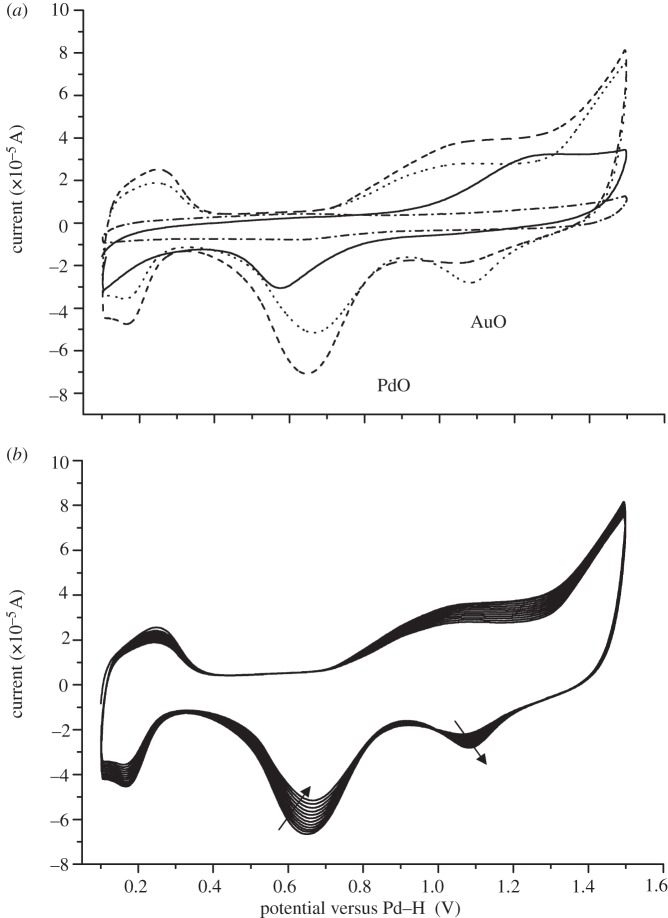
Surface analysis of bioPd–Au using cyclic voltammetry (CV). (*a*) Voltammetric profile of bioPd–Au in 0.1 M H_2_SO_4_ for the first cycle (solid line), the tenth cycle (dashed line), the last cycle (dotted line) and the glassy carbon support (dash-dotted line). Scan rate 100 mV s^−1^. (*b*) Voltammetric profile of bioPd–Au in 0.1 M H_2_SO_4_ from scan 10 onwards. The arrows show the increase/decrease of oxide peaks. Scan rate 100 mV s^−1^.

**Figure 3.**
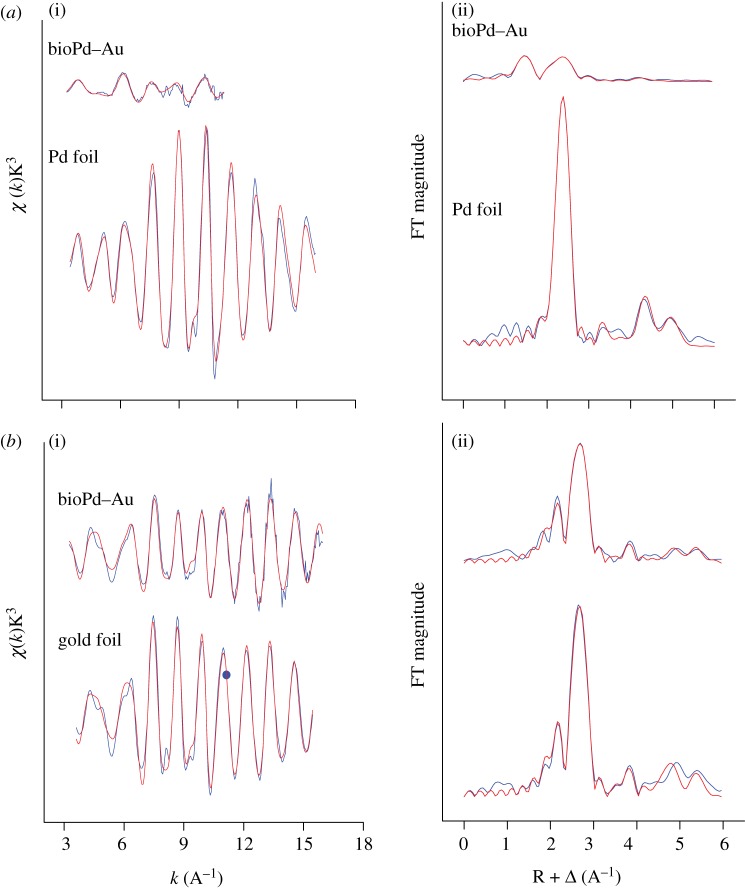
EXAFS analysis of bioPd–Au and reference compounds. *K*^3^-weighted EXAFS spectra (left panel) and corresponding FT (right panel) of bioPd–Au sample and reference compound at the (*a*) Pd K-edge and (*b*) Au L_III_-edge. Blue lines, data; red lines, fit.

Characterization of the obtained Pd–Au structures using X-ray absorption spectroscopy analysis shows an important degree of metal–metal coordination. Combined analysis of cyclic voltammetry (CV; figure 2) and EXAFS data obtained at the Pd K- and Au L_III_-edges (figure 3) is consistent with the development of a core/shell structure where surface-exposed Pd atoms decorate a core of Au atoms. Figure 2*a* shows the voltammetric profile for the bioPd–Au preparation. The first cycle shows the presence of Pd oxide and the absence of any Au oxide, i.e. most surface sites are occupied by Pd. Since XRD analysis (electronic supplementary material, figure S5) showed that Au occupied the bulk sites, the bioPd–Au NPs appear to exhibit an Au–Pd core/shell structure. Pd oxide desorption potentials correspond to those expected from bulk Pd although the full width at half maximum of the peak is somewhat larger than for bulk Pd [24], indicating that there is a significant perturbation brought about by the gold component of the NPs. Further potential cycling engenders changes to the surface associated with both oxidative cleaning of all Pd sites and Pd dissolution [24]. Au oxide stripping peaks are now visible and continue to increase in size as a function of potential cycling. This is completely consistent with continual electrochemical dissolution of Pd covering Au sites (after dissolution of a Pd capping layer). Interestingly, this is the reverse configuration of that predicted according to the sequence of reduction of the precursors, i.e. as Pd(0) seeds were used, surface Au-rich Pd NPs immobilized on cells (Pd core/Au shell NPs) were expected. Simple thermodynamic arguments, based on the lower surface energy of Au and stronger Pd–Pd bonding, would also favour the Pd_core_–Au_shell_ configuration [25]. This is clearly not the case here, as confirmed by CV (figure 2). Similar results have been reported in studies where the sacrificial hydrogen strategy was used to generate the Pd–Au NPs [22], where the mechanism was attributed to pre-formed Pd particles reducing Au(III) (the respective redox potentials are Au^3+^/Au, 1.002 V; Pd^2+^/Pd, 0.83V) to generate Pd^2+^ ions which then relocate around Au NPs and are reduced to Pd(0) via H_2_ on the Au–NP surface. The two NPs in figure 1*c* show different stages of this progression.

X-ray absorption near-edge structure analysis of the bioPd–Au sample at both Au and Pd edges shows that, while Au is mainly present as Au(0), Pd is present as a mixture of Pd(0) and Pd(II), with a significant dominance of the ionic part (electronic supplementary material, figure S6). The decrease of whiteline intensity observed at the Au L_III_-edge of the experimental sample in comparison with the Au foil sample is indicative of the decrease in the density of unoccupied sites of the Au 5d orbital in the bioPd–Au sample relative to the Au bulk, which is typical of Pd–Au alloy formation [26,27]. To confirm Pd–Au alloy formation and to discern different alloying motifs (random or core/shell-like non-random), EXAFS spectroscopy was used. Figure 3 shows the EXAFS spectra of bioPd–Au NPs and reference compounds (Au, Pd foils) at both the Pd K- and Au L_III_-edges (figure 3*a*(i), *b*(i)) along with their corresponding Fourier transforms (FTs; figure 3*a*(ii), *b*(ii)). Pd K and Au L_III_ structural parameters including the coordination number of the different paths (Au–Au, Au–Pd, Pd–Pd, Pd–Au and Au–M; Pd–M (where M is Au or Pd)) of Au, Pd foils and bioPd–Au NPs are summarized in table 1. The first shell Pd–metal coordination number (*N*_Pd−M_ = *N*_Pd−Au_ *+ N*_Pd−Pd_) was calculated to be 3.1 ± 0.4 (table 1) and is much smaller (by a factor of 3) than that of Au–metal (*N*_Au−M_ = *N*_Au−Au_ *+ N*_Au−Pd_) calculated to be 10.7 ± 0.6. According to Teng *et al.* [28], the fact that *N*_Pd−M_ < *N*_Au−M_ indicates that a larger number of Pd atoms segregate to the surface of the NPs and Au atoms are present in the core, since atoms on the surface have fewer neighbours than those in the core. In addition, the environment of Au atoms is highly ordered in the bioPd–Au sample, presumably owing to their preferential bonding in the core since no lattice expansion was observed in this sample as the *R*_Au−Au_ in Au foil and bioPd–Au were similar within the experimental errors. We conclude that the EXAFS fitting results are completely in accordance with a Pd shell and Au core structure as *N*(Au–M) ≫ *N*(Pd–M) (table 1). Additional evidence for the formation of a non-random alloy with a core/shell structure is the fact that the Au–Pd bond length (2.75 ± 0.02 Å) is smaller than those observed for Pd–Pd (2.76 ± 0.02 Å) and Au–Au (2.84 ± 0.02 Å). As suggested previously [28], Au–Pd bonds possibly formed very stable bridges between two sub-lattices at the interface of the ordered Au core and disordered Pd shell structure. The disorder in the Pd shell is due to the bonding of Pd atoms with O, as was demonstrated by EXAFS spectroscopy, which indicates that the Pd surface atoms are exposed/coordinated to oxygen and/or nitrogen donor atoms as significant contribution to the EXAFS signals arose from those of Pd–O or Pd–N bonds. EXAFS spectroscopy cannot distinguish between the Pd–O and Pd–N contribution; therefore, they are both modelled as Pd–O for simplicity. Thus, at the Pd edge, the first three peaks of the FT correspond to Pd–O_1_, Pd–O_2_ and Pd–Pd bonds, respectively. The distances were identified using the Pd–O and Pd–Pd backscattering phase and amplitude functions obtained from atomic coordinates of PdO using the FEFF 8 program. From the Pd–O coordination numbers, the fraction of oxidized atoms was estimated to be about 65% (1.2/1.9).

**Table 1.** Best-fit results obtained by EXAFS analysis of Pd foil, Au foil and bioPd–Al bimetallic sample.

sample	Pd foil	Au foil	bioPd–Au
*N*_Pd−Pd_	12^a^		1.9 (4)
*N*_Pd−Au_			1.2^b^
*N*_Au−Pd_			0.8 (2)
*N*_Au−Au_		12^a^	9.9 (6)
*N*_Pd−M_			3.1 (4)
*N*_Au−M_			10.7 (6)
^c^*N*_M−M_			7.8 (6)
*R*_Pd−Pd_ (Å)	2.74 (14)		2.76 (16)
*R*_Pd−Au_ (Å)			2.75 (17)
*R*_Au−Au_ (Å)		2.84 (19)	2.84 (18)
σ^2^_Pd−Pd_ (Å^2^)	0.0058 (58)		0.0060 (6)
σ*^2^*_Pd−Au_ (Å^2^)		0.0077 (72)	0.0039 (4)
σ^2^_Au−Au_ (Å^2^)			0.0088 (9)

^a^Value fixed for calculation.

^b^Coordination numbers were constrained in the fits to be varied in accordance with equation: *N*_Pd−Au_/*N*_Au−Pd_ = *X*_Au_/*X*_Pd_.

^c^*N*_M−M_ = *X*_Au_*N*_Au−M_ + *X*_Pd_*N*_Pd−M_ (where *X*_Au_ and *X*_Pd_ are the molar composition of Au and Pd, respectively).

The size of the bimetallic NPs, estimated by means of the determination of the average metal coordination number, *N*_M−M_, where *N*_M−M_ is 7.8 ± 0.6, corresponds to a particle size of 1.5–2.5 nm using a previously reported correlation between the coordination number and the particle size [29]*.* This is not consistent with the particle size estimation obtained from the XRD spectrum (estimated particle size of about 4.5 nm; see electronic supplementary material, figure S5), owing probably to the enhanced surface disorder (significant relaxation under the influence of ligands, e.g. oxygen donor atoms), which may result, according to Yevick & Frenkel [30], in the underestimation of particle size of metal NPs in the size range under 5 nm.

Finally, we showed the catalytic activity of the bioPd–Au NP material. A mass metal loading of 5% is commonly used for chemical catalysts; the NPs (2.5% Pd/2.5% Au) were reacted against benzyl alcohol. The biogenic catalyst in air compares well with the chemically prepared catalyst in O_2_ [14] (table 2; entry 3) at a similar catalyst loading. Much higher turnover frequencies (TOFs) can be achieved with TiO_2_-supported catalysts than thus far observed for the bioPd–Au catalyst, but these high activities appear to be favoured by much lower catalyst loadings (approx. 6 × 10^−5^ mol l^−1^ in entries 4–8, cf. approx. 130–180 × 10^−5^ mol l^−1^; entries 2 and 3). Also, entries 9 and 10 show a 20-fold enhancement in TOF over a 40°C increase in temperature, which suggests a high potential of the biomaterial as this has an activity comparable to that of entry 9 at a 30°C lower temperature.

**Table 2.** Comparison of the catalytic activity of the biocatalyst for benzyl alcohol oxidation with data from the literature.

no.	catalyst 2.5% Au/2.5%Pd on support as shown	reaction conditions						(metal) (10^−5^ mol l^−1^ alcohol)		TOF (h^−1^)^a^ at 0.5 h	reference
		catalyst (g)	alcohol (l)	*T* (K)	*P* (10^5^ Pa)	gas	*S* (r.p.m.)	Au	Pd		
1	*E. coli*	0.180	0.05	363	6	air	1200	44.6	82.5	1083	this study
2	*E. coli*	0.090	0.025	363	1	O_2_	1200	44.6	82.5	887	[31]
3	TiO_2_	0.200	0.04	373	2	O_2_	1500	63.5	118	607	[14]
4	TiO_2_	0.007	0.04	373	1	O_2_	1500	2.1	3.9	6190	[14]
5	TiO_2_	0.007	0.04	373	5	O_2_	1500	2.1	3.9	6190	[14]
6	TiO_2_	0.007	0.04	383	1	O_2_	1500	2.1	3.9	14 270	[14]
7	TiO_2_	0.007	0.04	433	1	O_2_	1500	2.1	3.9	86 500	[14]
8	TiO_2_	0.007	0.04	433	10	O_2_	1500	2.1	3.9	65 400	[32]
9	TiO_2_	0.025	0.04	393	10	O_2_	1500	7.9	14.7	1300	[33]
10	TiO_2_	0.025	0.04	433	10	O_2_	1500	7.9	14.7	28 400	[33]

^a^Calculation of turnover frequency (TOF, h^−1^) after 0.5 h of reaction. TOF is defined as molecules reacting per active site in unit time. Here, TOF numbers were calculated on the basis of the total loading of metals. P, pressure; S, stirrer speed.

## 4. Conclusion

In conclusion, we present a two-step biochemical and chemical hybrid route to produce ordered Au–Pd core/shell nanostructures. First, cells direct the formation of Pd NPs in the periplasm (via enzymatic reduction of Pd(II) precursors [11]), which are in turn used to seed the formation of Au NPs from solution. Pd(0) seeds reduce Au(III) and Pd relocates as a shell of Pd(0) via a Pd^2+^ intermediate under H_2_. The resulting biomass-supported Au–Pd core/shell NPs, and other bimetallics produced using this easy and scalable approach, could potentially find novel applications in diverse fields such as remediation of environmental pollutants [9], green chemistry/catalysis (e.g. selective oxidations) and be incorporated in proton exchange membrane fuel cells in lieu of the traditional Pt–Ru catalysts to enhance performances in a cost-efficient manner [10]. The catalytic activity of bioAu–Pd NPs in selective oxidations has recently been shown in our laboratory [31] and is reported here for benzyl alcohol oxidation in air at low temperature.
